# Acupuncture for Spinal Cord Injury and Its Complications: A Systematic Review and Meta-Analysis of Randomized Controlled Trials

**DOI:** 10.1155/2013/364216

**Published:** 2013-02-17

**Authors:** In Heo, Byung-Cheul Shin, Young-Dae Kim, Eui-Hyoung Hwang, Chang Woo Han, Kwang-Ho Heo

**Affiliations:** ^1^School of Korean Medicine, Pusan National University, Yangsan 626-870, Republic of Korea; ^2^Department of Rehabilitation Medicine, School of Korean Medicine, Pusan National University, Yangsan 626-870, Republic of Korea; ^3^Korean Medicine Hospital, Pusan National University, Yangsan 626-870, Republic of Korea

## Abstract

To evaluate the evidence supporting the effectiveness of acupuncture treatment for SCI and its complications, we conducted search across 19 electronic databases to find all of the randomized controlled trials (RCTs) that used acupuncture as a treatment for SCI and its complications. The methodological quality of each RCT was assessed using the Cochrane risk of bias tool and the PEDro scale. Sixteen RCTs, including 2 high-quality RCTs, met our inclusion criteria (8 for functional recovery from SCI, 6 for bladder dysfunction, and 2 for pain control). The meta-analysis showed positive results for the use of acupuncture combined with conventional treatments for the functional recovery in terms of motor ASIA scores and total FIM scores when compared to conventional treatments alone. Positive results were also obtained for the treatment of bladder dysfunction, in terms of the total efficacy rate, when comparing acupuncture to conventional treatments. However, 2 RCTs for pain control reported conflicting results. Our systematic review found encouraging albeit limited evidence for functional recovery, bladder dysfunction, and pain in SCI. However, to obtain stronger evidence without the drawbacks of trial design and the quality of studies, we recommend sham-controlled RCTs or comparative effectiveness research for each condition to test the effectiveness of acupuncture.

## 1. Introduction

Spinal cord injury (SCI) affects approximately 900 to 1,000 individuals per million in the general population, and it is estimated that there are 12,000 new cases of SCI every year in the United States [1]. Nearly 80% of the individuals who experience SCI are male, and, since 2000, the average age at injury has increased to 39.5 years, with 11.5% of those injured greater than 60 years of age [2]. These statistics indicate that SCI may be a significant social and economic burden for patients and their families.

SCI causes lesions, damaged neurological structures, and secondary pathophysiological changes in spared tissue [19]. Thus, complete or partial loss of sensory and motor function is the most significant result of injury, and most patients experience secondary complications, such as bladder and bowel dysfunction, chronic pain, infertility, and autonomic dysfunction [19].

There are many treatments for functional recovery from injury and the related complications that result from SCI; these include surgery [20], prescription drugs [21], behavioral therapy [22], physical therapy [23], and supportive treatment [24]. There are also a variety of treatments used for the secondary complications of SCI, such as intermittent catheterization for bladder dysfunction, analgesics for pain, and others [25]. These treatments tend to be administered over long periods of time, and because of the potential complications of treatment, there has been an increased interest in alternative medical treatments, including acupuncture and other related therapies (moxibustion and acupressure) [26]. Acupuncture based on Traditional Chinese Medicine has been commonly used for pain or neurological problems in Chinese cultures [27]. Additionally, many studies [28, 29] have analyzed the use of acupuncture for these types of problems; these studies report a variety of outcomes regarding level of function, pain, and quality of life.

Three recently published reviews have examined the results of studies that support the use of treatments such as acupuncture for individuals with SCI [26, 30, 31]. One review [30] that was limited to a Chinese literature suggested that acupuncture treatments have a positive effect, while a second [26] did not systematically evaluate the available evidence and even failed to include several important trials [3, 5–13, 15, 16, 18]. The third review was restricted to the pain related to SCI [31]. Therefore, the purpose of this systematic review was to provide a qualitative analysis of all of the randomized controlled trials (RCTs) to date that were designed to determine the effectiveness of acupuncture for patients with SCI and its complications along PRISMA guidelines [32].

## 2. Methods

### 2.1. Data Sources

This systematic review included studies published in electronic databases over the time period ranging from their inception to December 2011. Relevant publications were found in the following databases: the Cochrane Central Register of Controlled Trials (CENTRAL), the Cochrane Database of Systematic Review (CDSR), PubMed, MEDLINE, EMBASE, CINAHL, nine Korean databases (Korean Studies Information, DBPIA, Korea Institute of Science Technology Information, Korean National Assembly Library, RISS4U, KoreaMed, Korean medical database, Korean Traditional Knowledge Portal, and Oasis), Chinese database (China National Knowledge Infrastructure), and two Japanese databases (J-STAGE and JAMAS). In addition, we searched databases that contained registered trials, such as ClinicalTrials.gov (http://www.clinicaltrials.gov).

The search keywords used were (acupuncture OR acup∗ OR “electroacupuncture” OR “auricular acupuncture” OR “scalp acupuncture”) AND (“spinal cord injury” OR “spinal injury” OR “spinal cord trauma” OR “spinal cord lesion” OR “spinal cord damage” OR “spinal cord fracture” OR “spinal cord contusion”) in each base language. This search strategy was adjusted for each database. In addition, the bibliographies of relevant systematic reviews and clinical guidelines were manually searched. We also searched the gray literature that included dissertations, letters, government documents, research reports, conference proceedings, and abstracts when available. The reference section for each study was also searched. 

### 2.2. Study Selection

#### 2.2.1. Types of Studies

We evaluated RCTs that studied the clinical effects of acupuncture as a treatment for SCI or its direct complications. Dissertations and abstracts examining these topics were also included. The review included prospective, parallel RCTs, or cross-over RCTs that assessed the efficacy of acupuncture regardless of blinding, language, or the type of report. The title and abstract of each identified article were read by a single primary researcher (IH) who completed the screening process. If articles were not written in English, they were translated into English prior to screening. The articles that would then be potentially included in our analysis were carefully checked by 2 independent reviewers (IH, YDK).

#### 2.2.2. Types of Participants

The clinical trials included in our review examined patients with SCI or complications secondary to SCI. There were no restrictions related to the amount of time after injury, the type of injury, the site of injury, or participant's age.

#### 2.2.3. Types of Interventions

Our study considered the effects of needle acupuncture. We included needle acupuncture with or without electrical stimulation or heating by moxa, auricular acupuncture, and scalp acupuncture. We excluded injection acupuncture, nonneedling acupuncture (e.g., laser acupuncture), acupuncture-like intervention (e.g., acupressure, moxibustion), and mixed treatments. Control conditions in the reviewed studies included sham acupuncture, no treatment, and conventional treatment for SCI (e.g., medication, rehabilitation). Studies using cointerventions were included only if cointerventions were given to both treatment groups.

#### 2.2.4. Outcome Measures

The outcome measures we considered were neurologic status or score (i.e., the American Spinal Injury Association neurologic and functional score; the ASIA score), functional ability outcomes (i.e., the Fugl-Meyer score), activities of daily living (i.e., the Functional Independence Measure; FIM score), scores related to the efficacy rate (i.e., the rate of participants who demonstrated efficacy), and outcome measures related to the complications of SCI (i.e., the bladder function scale for bladder dysfunction, pain scores for the level of pain and range of motion; ROM). We also considered measures of general safety reported for acupuncture as a treatment.

### 2.3. Data Extraction and Quality Assessment

Two reviewers (IH, YDK) reviewed each article independently and were blinded to the findings of the other reviewer. The reviewers collected data relating to the methodology, outcome measures, results, and final conclusions of each study. The reviewers also quantitatively evaluated each study's methodological quality through the use of the Cochrane risk of bias [33] and the Physiotherapy Evidence Database (PEDro) scale [34], which was developed to assess the methodological quality of RCTs specifically pertaining to physical therapy interventions [35]. The PEDro scale allows researchers to assess the quality of studies based on a cutoff score: studies that score fewer than six points are considered to be “low quality,” while studies with a score equal to or greater than six points are considered to be “high quality” [36]. Any discrepancies between reviewers were resolved through discussion until a consensus was reached.

### 2.4. Data Synthesis

To summarize the effects of acupuncture on each outcome measure, we used Cochrane Collaboration software (Review Manager (RevMan) Version 5.1 for Windows. Copenhagen: The Nordic Cochrane Centre). We extrapolated the risk estimate (relative risk; RR) and the 95% confidence interval (CI) for dichotomous data. The standard mean difference (SMD) and the 95% CI were calculated for continuous data. The variance of the amount of change was imputed using a correlation factor of 0.4, which is the value suggested by the Cochrane Collaboration [33]. We pooled data across studies using random effect models if excessive statistical heterogeneity did not exist. The chi-squared test and the Higgins *I* ^2^ test were used to assess the heterogeneity of the data [33]. We planned to use a formal funnel plot to assess publication bias if more than 10 trials were included; however, we were unable to generate a funnel plot due to the small number of trials included in our meta-analysis. 

## 3. Results

### 3.1. Study Description

**Figure 1:**
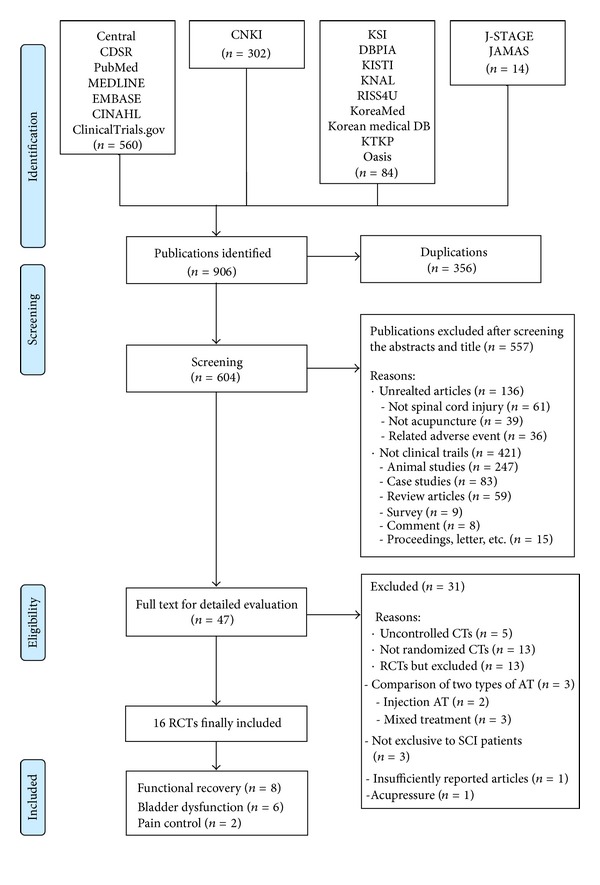
A flow chart describing the trial selection process. AT: acupuncture; CDSR: The Cochrane Database of Systematic Review; CENTRAL: The Cochrane Central Register of Controlled Trials; CNKI: China National Knowledge Infrastructure; CT: clinical trial; DB: database; KSI: Korean Studies Information; KISTI: Korea Institute of Science Technology Information; KNAL: Korean National Assembly Library; KTKP: Korean Traditional Knowledge Portal; RCT: randomized clinical trial; SCI: spinal cord injury.

We considered 960 potentially relevant articles. After screening the abstracts and titles, we excluded 557 studies (Figure 1). Forty-seven articles were fully evaluated. Thirty-one additional articles were subsequently excluded; 5 studies were not controlled and 13 studies were not randomized trials. Thirteen RCTs were also excluded: 3 of them compared 2 types of acupuncture [37–39], 2 analyzed injection acupuncture [40, 41], 3 included mixed treatments [42–44], 3 included patients other than those with SCI [45–47], 1 provided an insufficient report [48], and 1 used acupressure as opposed to acupuncture [49]. Consequently, 16 RCTs met our inclusion criteria [3–18]. Eight trials studied functional recovery in SCI (5 in traumatic SCI [4–6, 8, 10], 1 in nontraumatic SCI [7], and 2 were not reported [3, 9]), 6 trials studied bladder dysfunction secondary to SCI (1 in traumatic SCI [13], 1 in mixed SCI [15], and 4 were not reported [11, 12, 14, 16]), and 2 trials studied the use of acupuncture for pain control in SCI (2 trials did not report SCI type) [17, 18]. The main data are summarized in Table 1. There were 12 Chinese studies [3, 5–13, 15, 16], 2 Taiwanese studies [4, 14] and 2 American studies [17, 18]. Fifteen studies used 2-parallel-arm group designs [3–13, 15–18] and 1 used a 4-parallel-arm group design [14].

### 3.2. Study Quality

The mean PEDro score was 4.5; scores ranged from 4 to 8 points (Table 2). Only 2 of 16 RCTs met the PEDro criteria for high quality [9, 17]. The results of the Cochrane risk of bias analysis varied widely (Table 2). Two studies reported appropriate sequence generation [17, 18], in which the researchers used a coin toss method [18] and stratified block randomization [17]. Two of the trials reported inappropriate randomization procedures [8, 16], in which the researchers used hospital admission numbers for randomization. One trial blinded the participants and the assessors [17], and three blinded the assessors only [4, 9, 18]. Only one trial mentioned that the outcomes were analyzed with an intention-to-treat analysis [17]. Incomplete outcome data were addressed adequately in three studies [14, 17, 18]. Only one trial implemented allocation concealment; however, a detailed description of the method was not reported [9].

### 3.3. Descriptions of Acupuncture Treatment

The majority of the included RCTs stated that the rationale for acupuncture point selection was drawn from Traditional Chinese Medicine theory (Table 3). Fourteen studies used electrical stimulation with acupuncture [3–16] and 2 studies used needling acupuncture alone [17, 18]. Ten trials used fixed acupuncture points [4, 5, 7, 9–14, 16], 5 trials used fixed plus individualized acupuncture points [6, 8, 15, 17, 18], and 1 trial chose individualized by symptoms acupuncture points [3]. A total of 48 acupuncture points were included in functional recovery (meridian points: 36, extra point: 1, ear acupuncture points: 8, scalp acupuncture points: 2, Ashi point: 1), and 62.5% were located in the upper and lower extremities, 37.5% in head and back. A total of 18 acupuncture points were included in bladder dysfunction (meridian points: 17, extra point: 1) and 77.8% were located in the lower back. A total of 30 acupuncture points were included in pain control (meridian points: 29, Ashi point: 1) and 93.3% were located in shoulder area for the shoulder pain control of SCI.

### 3.4. Outcomes

#### 3.4.1. Functional Recovery

*Acupuncture versus Conventional Treatment (1 RCT)*

One RCT evaluated the effect of electrical acupuncture compared to rehabilitation [3]. A significant difference was found between the two groups regarding the total efficacy rate (*P* = 0.05, Table 1).

*Acupuncture Plus Conventional Treatment versus Conventional Treatment (7 RCTs)*

Five RCTs evaluated the effects of electrical acupuncture plus rehabilitation and compared the results to those of rehabilitation alone [4–7, 9]. Three RCTs described improved outcomes with electrical acupuncture plus rehabilitation over rehabilitation alone [4, 7, 9]. However, 2 RCTs did not show any significant differences between treatment groups (Table 1) [5, 6].

One RCT compared the effects of electrical acupuncture, rehabilitation, and oral neurotropic drugs to those of rehabilitation and oral neurotropic drugs [8]. This study showed a positive effect in terms of rehabilitation effectiveness only (*P* = 0.05, Table 1). 

One RCT evaluated the effects of electrical acupuncture plus intravenous drugs compared to intravenous drugs alone [10]. This RCT showed that treatment consisting of electrical acupuncture plus intravenous drugs was more effective than intravenous drugs alone (*P* = 0.003). 

**Figure 2:**
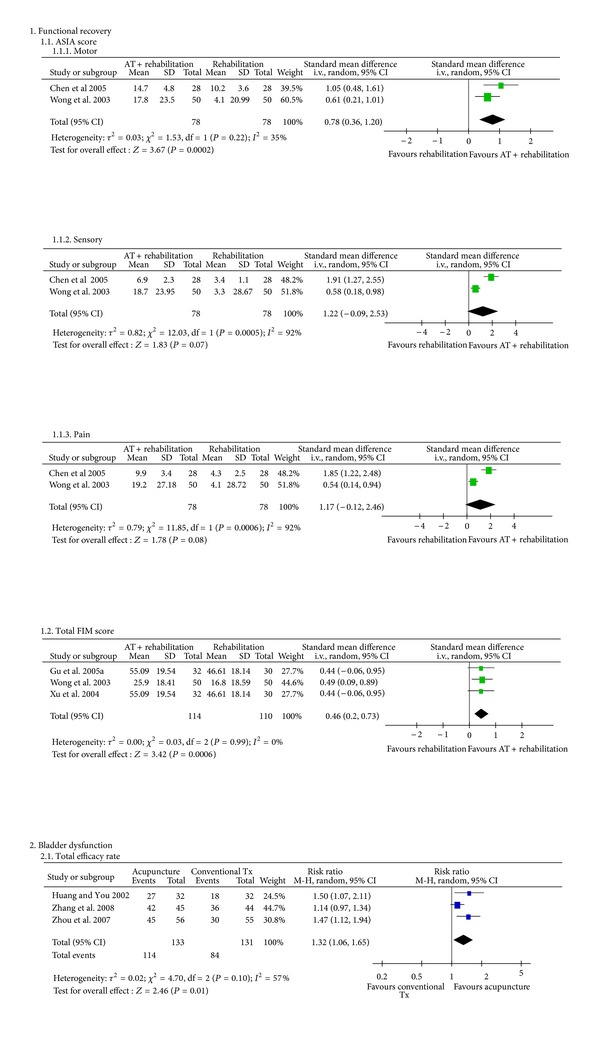
The meta-analysis of acupuncture for spinal cord injury and its complications. ASIA: American Spinal Injury Association; AT: acupuncture; FIM: functional independence measure; Tx: treatment.

The meta-analysis of acupuncture plus conventional treatment versus conventional treatment alone showed that treatment including acupuncture led to significantly more improved motor ASIA scores (2 studies [4, 7]: *n* = 156, SMD = 0.78, 95% CI of 0.36 to 1.20, *P* = 0.0002, heterogeneity: *χ* ^2^ = 1.53, *P* = 0.22, and *I* ^2^ = 35%; Figure 2) and total FIM scores (3 studies [4, 6, 8]: *n* = 224, SMD = 0.46, 95% CI of 0.20 to 0.73, *P* = 0.0006, heterogeneity: *χ* ^2^ = 0.03, *P* = 0.99, and *I* ^2^ = 0%; Figure 2).

#### 3.4.2. Bladder Dysfunction

*Acupuncture versus Conventional Treatment (3 RCTs)*

Three RCTs evaluated the effects of electrical acupuncture compared to conventional treatment (intramuscular neostigmine methylsulfate, intermittent catheterization, bladder training, etc.) [11–13].

Two of the 3 RCTs used intramuscular neostigmine methylsulfate plus intermittent catheterization and bladder training [11, 12]. One study showed that electrical acupuncture induced significantly greater improvement when compared to conventional treatment alone in terms of the total efficacy rate (*P* = 0.02, Table 1) [11]. However, the other study did not show a significant difference between treatment conditions (*P* = 0.11, Table 1) [12].

One of the 3 RCTs compared the effects of electrical acupuncture with conventional treatment (intermittent catheterization and bladder training) [13]. This study showed a significant positive effect for electrical acupuncture in terms of the total efficacy rate (*P* = 0.006, Table 1) and the levels of residual urine (*P* < 0.00001, Table 1) [13]. 

The meta-analysis of acupuncture versus conventional treatment showed a significant positive effect of acupuncture in terms of the total efficacy rate (3 studies [11–13]: *n* = 264, RR= 1.32, 95% CI of 1.06 to 1.65, *P* = 0.01, heterogeneity: *χ* ^2^ = 4.70, *P* = 0.10, and *I* ^2^ = 57%; Figure 2).

*Acupuncture plus Conventional Treatment versus Conventional Treatment Alone (3 RCTs)*

Two RCTs compared the effects of electrical acupuncture plus intermittent catheterization and bladder training to intermittent catheterization and bladder training alone [14, 16]. A recalculation of the mean difference (MD) revealed that there was a significant positive effect for electrical acupuncture in terms of the total days needed to reach bladder balance (*P* < 0.05, Table 1) [14]; however, there was not a positive effect in bladder voiding function parameters (the frequency of urination, the maximum voided volume, bladder capacity, residual urine, and quality of life scores, Table 1) [16]. 

One RCT compared the effects of electrical acupuncture plus intermittent catheterization versus intermittent catheterization alone [15]. This study showed a positive effect in terms of the total efficacy rate (*P* = 0.007, Table 1) [15].

#### 3.4.3. Pain Control 

*Acupuncture versus Sham Acupuncture (1 RCT)*

 One RCT evaluated the effects of manual acupuncture with no manipulation at sites located at least 1 cun (1 Chinese anatomic inch, approximately 2.5 cm) away from the established meridian and extra points [17]. The results showed that acupuncture did not significantly affect the PU-WUSPI and the NRS (Table 1) [17].

*Acupuncture versus Trager Approach (1 RCT)*

 One RCT assessed the effects of manual acupuncture compared to the Trager Approach (a form of bodywork and movement reeducation developed by Milton Trager) [18]. Our recalculation of the MD showed that there was no significant difference between the 2 treatments (*P* > 0.05, Table 1) [18].

#### 3.4.4. The Safety Reporting of Acupuncture

Only three RCTs reported adverse events associated with acupuncture [7, 17, 18]. Two RCTs reported no adverse events [7, 18], and 1 trial reported minimal adverse effects without a detailed explanation [17].

## 4. Discussion

This is the first systematic review and meta-analysis of RCTs that fully evaluates the effectiveness of needle acupuncture for SCI and its complications. Of the 16 trials included in this paper, 8 trials studied the effects of acupuncture on functional recovery in SCI [3–10] and 8 trials that studied its effect on the secondary complications that follow SCI (6 trials for bladder dysfunction [11–16], and 2 trials for pain levels [17, 18]). 

Based on the Cochrane risk of bias [33] and the PEDro scale [34], the methodological quality and design of the primary studies was mostly poor (only 2 of 16 were considered high quality, 12.5%). Of the 16 studies included in our review, 2 trials used appropriate sequence generation methods [17, 18]. Only one of the studies reported allocation concealment [9]. One RCT reported details of assessor- and patient-blinding procedures [17]. Trials with inappropriate randomization were threatened by selection bias [50] and inadequate blinding tended to exaggerate the effects of treatment [33]. Researchers should conduct their trials according to the CONSORT statement [51]. In addition, 14 of the 16 RCTs originated from Chinese sources [3–16]. Several groups have shown that the majority of Chinese acupuncture studies report positive results [52, 53]. Therefore, it is possible that a publication bias exists; although we searched extensively for all the studies that are relevant to this paper, we may have failed to conduct an analysis of publication bias [54]. This phenomenon casts a considerable doubt on the reliability of these data.

Ten trials compared acupuncture plus various conventional therapies to conventional therapies alone [4–10, 14–16], and 5 trials compared acupuncture to conventional therapies [3, 11–13, 18]. Such trial designs are open to bias because participants were not blinded. Sham-controlled trials could control nonspecific effects of acupuncture [55]. Only one trial adopted a penetrating sham control [17]. This sham-controlled trial had a small sample size, and our recalculated power for this study was 0.326. This finding indicates that the study lacked sufficient statistical power. Thus, these results limited our ability to evaluate the effectiveness of this treatment for various conditions in SCI. Additionally, nonpenetrating sham acupuncture may be more acceptable than penetrating sham [56, 57].

Although the effectiveness of acupuncture is unclear in SCI or its complications, acupuncture therapy is a relatively convenient and safe treatment for some conditions [58]. SCI patients who receive long-term rehabilitation or medication treatments may occasionally need a safe nonpharmacological treatment [59]. In this regard, acupuncture can be useful to treat SCI and its complications if patients experience side effects or have no (or a weak) response to a conventional treatment. Thus, we carefully present several points that must be discussed in future research.

First, although several animal studies of SCI have reported that acupuncture induces neuronal function recovery [60], an analgesic effect [61], and anti-inflammatory responses [62], the RCTs included in this paper might have used specific treatment conditions that may not have fully drawn the maximal benefit from acupuncture. To evaluate the effects of acupuncture on functional recovery in SCI, an appropriate set of treatment conditions which can maximize the therapeutic effect of acupuncture is highly recommended. Also, the conditions have to include the appropriate duration of treatment resulted from comparison of duration of the course of acupuncture treatment. Acupuncture as a (sole or adjunct) treatment for bladder dysfunction demonstrated a positive effect over all conventional treatments except one; while this is encouraging, this result is inconclusive due to the small sample size and low methodological quality. The sham trial for pain control that was reviewed failed to show any specific positive analgesic effect of acupuncture; however, this trial lacked statistical power. It is possible that acupuncture is an effective treatment for pain in SCI, as indicated by a previous rigorous review [31]. Thus, future high-quality trials on secondary conditions of SCI should be conducted to evaluate the potential effects of acupuncture [63].

Second, the prescription of acupuncture points was not consistent across studies (Table 3). Also, the degree of stimulation and the duration and frequency of acupuncture treatment were very various (Table 3). And we could not find the consistency of the Chinese medicine pattern in included trials. So it was difficult to estimate the correlation between the difference of acupuncture treatment and its therapeutic effectiveness. They might not have been optimal or might even be considered an underdosage of treatment for SCI [64]. As a matter of fact, the optimal prescription of acupuncture treatment (acupuncture point, degree of stimulation, frequency of treatment, and a number of treatment sessions) for SCI is a controversial issue amongst acupuncture experts. A standardized prescription of acupuncture for SCI or its complications is necessary.

Third, sham controls in acupuncture research for SCI may represent an ethical issue [65]. Therefore, an adjunctive treatment of acupuncture to conventional treatments for SCI can be evaluated by RCTs comparing acupuncture plus conventional treatment to sham acupuncture plus conventional treatment or through comparative effectiveness research (CER) [66].

## 5. Conclusion

The results of our systematic review and meta-analysis suggest that the evidence for the effectiveness of acupuncture as a symptomatic treatment for SCI and its complications is encouraging but limited. There is a great need to test the clinical implications of acupuncture for a number of SCI-related conditions. The efficacy of acupuncture for SCI and its complications must be studied using sham-controlled RCTs or CER with a standardized acupuncture procedure; such RCTs would conform to the recommendations of the CONSORT and STRICTA guidelines [51, 67].

## Figures and Tables

**Table 1:** A summary of the randomized controlled trials of acupuncture for spinal cord injury.

First author (ref) (year) Country	Study design	Patient population Type of SCI Target state Duration of SCI (mean (range))	Experimental treatment (regimen)	Control treatment (regimen)	Main outcomes	Intergroup differences Experimental versus control
Functional recovery						
Chen [3] (1995), China	Parallel 2 arms	67 n.r. Lower extremity spasticity (A) 11.3 ± 10.0 (1–53) mo (B) 15.5 ± 16.7 (1–81) mo	(A) EA, (*n* = 32)	(B) Rehabilitation, (*n* = 35)	Total efficacy rate (Ashworth scale)	RR; *P* = 0.05, 1.86 (1.00, 3.45)
Wong [4] (2003), Taiwan	Parallel 2 arms Assessor blind	100 Traumatic SCI Complete motor paralysis n.r.	(A) EA + AA, plus (B), (*n* = 50)	(B) Rehabilitation, (*n* = 50)	(1) ASIA score (1) Motor (2) Sensory (3) Pain (2) Total FIM score	(1) (1) MD, *P* = 0.003, 0.61 (0.21, 1.01) (2) MD, *P* = 0.005, 0.58 (0.18, 0.98) (3) MD, *P* = 0.009, 0.54 (0.14, 0.94) (2) MD, *P* = 0.02, 0.49 (0.09, 0.89)
Cui [5] (2004), China	Parallel 2 arms	72 Traumatic SCI n.r. (A) 12.9 ± 5.1 d (B) 13.4 ± 6.2 d	(A) EA, plus (B), (*n* = 37)	(B) Rehabilitation, (*n* = 35)	(1) FIM score (complete independent rate) (1) 3 sessions (2) 6 sessions	(1) (1) RR, *P* = 0.36, 2.84 (0.31, 26.01) (2) RR, *P* = 0.34, 1.89 (0.51, 6.99)
Xu [6] (2004), China	Parallel 2 arms	62 Traumatic SCI n.r. n.r.	(A) EA, plus (B), (*n* = 32)	(B) Rehabilitation, (*n* = 30)	Total FIM score	MD, *P* = 0.08, 0.44 (−0.06, 0.95)
Chen [7] (2005), China	Parallel 2 arms	56 Nontraumatic SCI Acute SCI n.r.	(A) EA + AA, plus (B), (*n* = 28)	(B) Rehabilitation, (*n* = 28)	(1) ASIA score (1) Motor (2) Sensory (3) Pain (2) FIM score (locomotion ability)	(1) (1) MD, *P* = 0.0003, 1.05 (0.48, 1.61) (2) MD, *P* < 0.00001, 1.91 (1.27, 2.55) (3) MD, *P* < 0.00001, 1.85 (1.22, 2.48) (2) MD, *P* < 0.00001, 2.13 (1.46, 2.79)*
Gu [8] (2005)a, China	Parallel 2 arms	62 Traumatic SCI n.r. (A) 30.3 ± 17.6 d (B) 28.8 ± 11.7 d	(A) EA, plus (B), (*n* = 32)	(B) Rehabilitation + neurotropic oral drugs, (*n* = 30)	(1) Total FIM score (2) Rehabilitation effectiveness (= (FIM discharge − FIM admission)/hospitalization day)	(1) MD, *P* = 0.08, 0.44 (−0.06, 0.95) (2) MD, *P* = 0.05, 0.51 (0.01, 1.02)
Ma [9] (2005), China	Parallel 2 arms Assessor blind	30 n.r. SCI (walking function) n.r.	(A) EA + AT, plus (B), (*n* = 15)	(B) Rehabilitation, (*n* = 15)	(1) Fugl-Meyer's score (2) Lindmark's score	(1) MD, *P* = 0.008, 1.04 (0.27, 1.81) (2) MD, *P* < 0.00001, 8.55 (6.12, 10.98)
Sheng [10] (2009), China	Parallel 2 arms	48 Traumatic SCI n.r. n.r.	(A) EA, plus (B), (*n* = 24)	(B) IV (BPH 120 mg + 0.9% NaCl 250 mL, daily for 3 months), (*n* = 24)	(1) Total efficacy rate	(1) RR, *P* = 0.003, 2.10 (1.28, 3.45)

Bladder dysfunction						
Huang [11] (2002), China	Parallel 2 arms	64 n.r. Urinary retention (A) 11.0 (5–20) d (B) 10.5 (5–20) d	(A) EA, (*n* = 32)	(B) IM (Neostigmine methylsulfate, (1 mg/2 mL), 2 hours after catheter removal) + IC + BT (*n* = 32)	Total efficacy rate	RR, *P* = 0.02, 1.50 (1.07, 2.11)
Zhang [12] (2008), China	Parallel 2 arms	89 n.r. Neurogenic bladder (A) 2~3 m (B) n.r.	(A) EA, (*n* = 45)	(B) IM (Neostigmine 0.5~1 mg, once a day) + IC + BT, (*n* = 44)	Total efficacy rate	RR, *P* = 0.11, 1.12 (0.95, 1.32)
Zhou [13] (2007), China	Parallel 2 arms	111 Traumatic SCI Neurogenic bladder (A) 45.62 ± 6.23 d (B) 43.76 ± 8.23 d	(A) EA (*n* = 56)	(B) IC + BT, (*n* = 55)	(1) Total efficacy rate (2) Residual urine (mL)	(1) RR, *P* = 0.006, 1.47 (1.12, 1.94) (2) MD, *P* < 0.00001, −1.16 (−1.56, −0.76)
Cheng [14] (1998), Taiwan	Parallel 4 arms	80^†^ n.r. Neurogenic bladder (A) 23.7 ± 12.8 d (B) 26.1 ± 12.1 d	(A) EA, plus (B) (1) above T11 (*n* = 18) (2) below T11 (*n* = 14)	(B) IC + BT, (1) above T11 (*n* = 16) (2) below T11 (*n* = 12)	Total days needed to reach bladder balance (1) Above T11 (2) Below T11	(1) MD, *P* = 0.003, 1.10 (0.37, 1.83) (2) MD, *P* = 0.009, 1.12 (0.28, 1.96)
Gu [15] (2005)b, China	Parallel 2 arms	64 mixed Bladder dysfunction n.r.	(A) EA, plus (B), (*n* = 32)	(B) IC, (*n* = 32)	Total efficacy rate	RR, *P* = 0.007, 1.53 (1.12, 2.08)
Liu [16] (2009), China	Parallel 2 arms	40 n.r. Bladder dysfunction 14 days~90 d	(A) EA, plus (B), (*n* = 20)	(B) IC + BT, (*n* = 20)	Bladder voiding function parameters (1) Frequency of urination (times) (2) Maximum voided volume (mL) (3) Bladder capacity (mL) (4) Residual urine (mL) (5) Quality of life score	(1) MD, *P* = 0.012, −0.49 (−1.12, 0.14) (2) MD, *P* = 0.024, 0.37 (−0.25, 1.00) (3) MD, *P* = 0.06, 0.61 (−0.02, 1.25) (4) MD, *P* = 0.32, −0.32 (−0.94, 0.31) (5) MD, *P* = 0.30, −0.33 (−0.96, 0.29)

Pain condition						
Dyson-Hudson [17] (2007), USA	Parallel 2 arms Patient blind Assessor blind	17 n.r. Chronic shoulder pain (A) 9.3 ± 10.5 y (B) 13.1 ± 7.7 y	(A) AT, (*n* = 8)	(B) Sham AT, (*n* = 9)	(1) PC-WUSPI (2) NRS (11 points scale, shoulder pain)	(1) MD, *P* = 0.19, −0.65 (−1.64, 0.33) (2) MD, *P* = 0.19, −0.67 (−1.69, 0.34)
Dyson-Hudson [18] (2001), USA	Parallel 2 arms	18 n.r. Chronic SCI and shoulder pain (A) 16.2 ± 9.7 y (B) 13.4 ± 6.2 y	(A) AT, (*n* = 9)	(B) Trager Approach, (*n* = 9)	(1) PC-WUSPI (2) NRS (10 points VAS, shoulder pain) (3) VRS (shoulder pain)	(1) MD, *P* = 0.91, −0.05 (−0.98, 0.87) (2) No significant difference (*P* > 0.05)^‡^ (3) RR, *P* = 0.46, 0.89 (0.67, 1.20)

AA: auricular acupuncture; ASIA score: American spinal injury association neurologic and functional score; AT: acupuncture; BPH: brain protein hydrolysate; BT: bladder training; EA: electrical AT; FIM score: functional independence measure score; IC: intermittent catheterization; IM: intramuscular; IV: intravenous; n.r.: not reported; NRS: numeric rating scale; PC-WUSPI score: performance-corrected wheelchair user's shoulder pain index score; SCI: spinal cord injury; VAS: visual analog scale; VRS: verbal response score.

*The author did not report total FIM score but did each of 6 domains.

^†^80 patients were randomized, but 60 were analyzed.

^‡^Each group had a significant effect after therapy (*P* < 0.01).

**Table 2:** Quality assessment of included randomized clinical trials.

First author (year)	PEDro scale item												Cochrane risk of bias							
	A	B	C	D	E	F	G	H	I	J	K	Total	L	M	N			O	P	Q
															Patient	Therapists	Assessors
Chen [3] (1995)	1	1	0	0	0	0	0	1	0	1	1	4	U	U	N	N	U	U	U	N
Wong [4] (2003)	1	1	0	0	0	0	1	1	0	1	1	5	U	U	N	N	Y	U	U	N
Cui [5] (2004)	1	1	0	1	0	0	0	1	0	1	1	5	U	U	N	N	U	U	U	N
Xu [6] (2004)	1	1	0	0	0	0	0	1	0	1	1	4	U	U	N	N	U	U	U	N
Chen [7] (2005)	1	1	0	0	0	0	0	1	0	1	1	4	U	U	N	N	U	U	U	N
Gu [8] (2005)a	1	1	0	1	0	0	0	1	0	1	1	5	N	U	N	N	U	U	U	N
Ma [9] (2005)	1	1	1	1	0	0	1	1	0	1	1	7	U	Y	N	N	Y	U	U	N
Sheng [10] (2009)	1	1	0	0	0	0	0	1	0	1	1	4	U	U	N	N	U	U	U	N
Huang [11] (2002)	1	1	0	1	0	0	0	1	0	1	1	5	U	U	N	N	U	U	U	N
Zhang [12] (2008)	1	1	0	1	0	0	0	0	0	1	1	4	U	U	N	N	U	U	U	N
Zhou [13] (2007)	1	1	0	1	0	0	0	0	0	1	1	4	U	U	N	N	U	U	U	N
Cheng [14] (1998)	1	1	0	1	0	0	0	0	0	1	1	4	U	U	N	N	U	Y	U	U
Gu [15] (2005)b	1	1	0	0	0	0	0	1	0	1	1	4	U	U	N	N	U	U	U	N
Liu [16] (2009)	1	1	0	0	0	0	0	1	0	1	1	4	N	U	N	N	U	U	U	N
Dyson-Hudson [17] (2007)	1	1	0	1	1	0	1	1	1	1	1	8	Y	U	Y	N	Y	Y	Y	N
Dyson-Hudson [18] (2001)	1	1	0	0	0	0	1	1	0	1	1	5	Y	U	N	N	Y	Y	U	N

PEDro scale items (each satisfied item except the first item contributes 1 point to the total PEDro score): A: eligibility criteria specified, B: randomization, C: allocation concealment, D: groups similar at baseline, E: blinded subjects, F: blinded therapist, G: blinded assessors, H: adequacy of followup, I: ITT analysis, J: between-group comparison, K: point and variability measures; 1: item positive, 0: item negative or unknown.

Cochrane risk of bias: L: was the allocation sequence adequately generated? M: was allocation adequately concealed? N: was knowledge of the allocated intervention adequately prevented during the study? O: were incomplete outcome data adequately addressed? P: are reports of the study free of suggestion of selective outcome reporting? Q: was the study apparently free of other problems that could put it at a high risk of bias? Yes (Y): low risk of bias, no (N): high risk of bias, and unclear (U): uncertain risk of bias.

**Table 3:** Summary of treatment acupuncture points and other information related to acupuncture.

First author (ref) (year)	Acupuncture method	Regime	Acupuncture points	Deqi	Rationales for acupuncture points	Number of CMSP	Adverse events
Functional recovery							
Chen [3] (1995)	Individualized by injured spinal level	EA (1-2 Hz, 1 session = once a day, 30 min, 6 times a week, total 48 treatments)	Injured spinal level, upper 1 point and lower 1 point of Governer vessel of injured level (inter spinous process)	n.r.	TCM theory	1	n.r.
Wong [4] (2003)	Fixed	EA + AA (75 Hz, 10 mV, 1 session = 30 min, 5 times a week, till discharge)	EA: bilateral SI3, BL62 AA: 4 acupoints related to the spinal cord at the antihelix, helix, and lower portion of the ear-back areas	Considered	n.r.	1	n.r.
Cui [5] (2004)	Fixed	EA (1 session = once a day, 30 min for 1 month, total 90 or 180 treatments)	Arm: HT1, LU5, PC3, HT3 Leg: SP12, BL37 or GB30, BL40 Other peroneal nerve stimulation point (no reports about where EA was applied)	Considered	TCM theory	1	n.r.
Xu [6] (2004)	Fixed + individualized by symptoms	EA (1 Hz, 1 session = once a day, 30 min for 1 month, 7 days' rest, total 150 treatments)	EX-B2 + LI4, LI11, LI15, TE5, GB30, GB31, GB34, GB39, ST36, ST41, BL60, LR3 (limb dyskinesia) or SP6, SP9, BL25, eight-*liao* (evacuation disorder)	n.r.	TCM theory	2	n.r.
Chen [7] (2005)	Fixed	EA + AA (EA; 1–5 Hz, 1 session = once a day, 30 min, 6 days a week for 3 months, session interval 1-2 weeks, till discharge, AA; 1 session = once a day, 10 times, total 2-3 session, each ear alternately)	EA: bilateral SI3, BL62 AA: brain, subcortex, sympathetic, Shenmen	Impossible in SCI patients because of sensory impairment (+)	TCM theory	1	No adverse events (+)
Gu [8] (2005)a	Fixed + individualized by symptoms	EA (1 Hz, 3–5 V, 1 session = once a day, 30 min for 1 month, 1 week rest, total 150 treatments)	EX-B2, LI15, LI11, TE5, LI4, GB30, GB31, GB34, ST36, GB39, BL60, ST41, LR3 (EA applied to major extremity points) + eight-*liao*, BL25, SP6, GB34 (evacuation disorders)	n.r.	TCM theory	2	n.r.
Ma [9] (2005)	Fixed	EA + AT (scalp EA; 5 min, body AT; 25 min, once a day, total 168 sessions)	Scalp EA: MS6 (motor area), MS14 (equilibrium area) Body AT: LI10, LI11, TE8, SI5 (arm), LR12, GB34, GB39, BL54, BL60, LR3 (leg)	Considered (authors did not describe, but might be considered)	TCM theory	1	n.r.
Sheng [10] (2009)	Fixed	EA (1 session = once a day, 30 min for 10 days, 2 days' rest, total 70 treatments)	EX-B2, LI4, LI11, LI15, TE5, ST31, ST32, ST36, GB34	n.r	TCM theory	1	n.r.

Bladder dysfunction							
Huang [11] (2002)	Fixed	EA (continuous wave, 1 session = 30 min, for 5 days, 3 days' rest, total 5–20 treatments)	Bilateral BL54, ST28, BL32, BL34, T12-L2 Huatuojiaji (EX-B2), SP9, SP6	Considered (authors did not describe, but might be considered)	TCM theory	1	n.r.
Zhang [12] (2008)	Fixed	EA (2 Hz, 6 V, 1 session = once a day, 30 min for 2 weeks, total 12 treatments)	Bilateral BL23, BL35	n.r.	TCM theory	1	n.r.
Zhou [13] (2007)	Fixed	EA (continuous wave, 80 Hz, 20 mA, 1 session = once a day for 15 days, 5 days' rest, total 30 treatments)	Bilateral BL31, BL32, BL33, BL34, BL35	Considered	TCM theory	1	n.r.
Cheng [14] (1998)	Fixed	EA (20–30 Hz, 30–50 mA, 1 session = 15 min, 4~5 sessions each week, till their bladders were balanced)	CV3, CV4, BL32 (bilateral)	Considered	TCM theory	2	n.r.
Gu [15] (2005)b	Fixed + individualized by symptoms	EA (continuous wave, 1 session = once a day, 30 min for 2 weeks, total 14–56 treatments)	eight-*liao* (EA) + BL23, CV6, BL20, BL22 (deficiency syndrome) or SP6, BL28, SP9, CV3 (excess syndrome)	Considered	TCM theory	1	n.r.
Liu [16] (2009)	Fixed	EA (1 session = once a day, 30 min for 15 days, total 60 treatments)	EX-B2	n.r.	TCM theory	1	n.r.

Pain condition							
Dyson-Hudson [17] (2007)	Fixed + individualized by symptoms	AT (1 session = 20 min, twice a week, total 10 treatments)	Local points (chosen 6 points according to shoulder pain symptoms): LI14, LI15, LI16, TE13, TE14, TE15, GB21, SI9, SI10, SI11, SI12, SI13, SI14, SI15, LU1, LU2, PC2 Distal points (chosen 2 points according to local points used): LI2, LI4, LI10, LI11, LI18, SJ3, SI6, LU3, GV14, GB20, BL 10, BL11 Ashi points (1~4 points per treatment)	Considered	TCM theory	1	Minimal adverse effect
Dyson-Hudson [18] (2001)	Fixed + individualized by symptoms	AT (1 session = 20~30 min, twice a week, total 10 treatments)	Local points (chosen 6 points according to shoulder pain symptoms): LI14, LI15, LI16, TE13, TE14, TE15, GB21, SI9, SI10, SI11, SI12, SI13, SI14, SI15, LU1, LU2, PC2 Distal points (chosen 2 points according to local points used): LI2, LI4, LI10, LI11, LI18, SJ3, SI6, LU3, GV14, GB20, BL 10, BL11 Ashi points (1~4 points per treatment)	Considered	TCM theory	1	No adverse events (+)

AA: auricular acupuncture; AT: acupuncture; CMSP: Chinese medicine syndrome pattern; EA: electrical AT; n.r.: not reported; TCM: traditional Chinese medicine; (+): mentioned in text.
